# On the Use of Potash in Some Cutaneous Diseases

**Published:** 1859-11

**Authors:** James C. White


*On the Use of Potash in some Cutaneous Diseases.* By JAMES C. WHITE, M. D. Read before the Boston Society for Medical Observation, October 3d, 1859, and communicated for the Boston Medical and Surgical Journal.

It may be seen by the microscope that a drop of a solution of an alkali, when in contact with animal tissues, causes their solution and disappearance. The same effect is produced if we apply caustic potash to the living skin, the fatty tissues being saponified, and the albuminoid principles forming also definite chemical compounds, which are soluble in the excess of serous fluid poured out under the influence of this stimulus. Hence its caustic properties, so valuable when portions of living tissues are to be destroyed, and the knife may not be used. Of potash, however, in its dry form, or as Vienna paste, we do not intend to speak, but to consider its use in solution, by which the severity of its action may be exactly controlled and delicately graduated, or in the form of sapo viridis applied externally in the treatment of certain affections of the skin.

The application of the stronger solutions of potash, one part to two of water for instance, to the living skin, acts as a proper caustic, destroying the superficial layers of epidermis, and producing an abundant liberarian of fluid in which the albuminate of potash and soapy matter are found dissolved. The skin, under its action, looks as if its sweat-glands were working vigorously, like the forehead on a summer's day. This same drastic action of many of the potash salts on the mucous membrane, explains their cathartic and diuretic effect when given internally. The lymph, which is poured out over the raw surface, soon hardens and forms a thin pellicle like collodion, beneath which granulations goes on, protected from the free air. The weaker solutions and the soap fortunately do not act vigorously upon the healthy skin, but in a most discriminating manner affect the dried and diseased tissues.

*Sapo viridis*, or schmier-seife, plays a most important part in the treatment of cutaneous diseases in Germany, in hospital and private practice. Especially does Hebra, professor in the department of cutaneous diseases in the Vienna School, and who is about to publish a work on their pathology and treatment, which in point of magnificence and extent will far surpass any medical work ever published; especially does Hebra show its efficacy in many of the many cases which make his clinique so celebrated. It is prepared by boiling fish or other animal oils with an excess of lye composed of caustic potash and the crude carbornate. It varies in color and purity, according to the ingredients and mode employed in its manufacture, and as found in commerce is often of a dark green or black color. The present specimen, obtained of L. Babo, German apothecary, 311 Tremont Street, is a first-rate article, and contains no free potash, which secures its even action upon the skin, and prevents the excoriations which sometimes follows the violent inunction of this remedy when alkali exists in an uncombined state. The best preparations have a bright amber or green color, a uniformly soft consistence, and a strong odor of potash. Rubbed upon the healthy skin, it produces a slight reddening only, but if the friction be continued a long time and vigorously pushed, excoriations and various eruptive appearances (as miliaria, urticaria and eczema) may present themselves.

The affections of the skin in which these alkaline preparations are most useful, are the following: molluscum contagiosum, or seborrhœa; acne; eczema; scabies; prurigo; psoriasis; pityriasis versicolor. When the openings of the sebaceous glands are stopped, we very often find a plug of sebum distending the duct and mouth, which, acting as a foreign body, produces inflammation of the gland and surrounding skin. This is followed by degeneration of the follicles, and forms the disease called seborrhœa, or strophulus albidus of Willan. These comedones are most often met with on the nose, and affect principally persons of a gross habit. Not unfrequently many such diseased follicles unite to form a single tumor, from which exudes a milky fluid. This is the molluscum contagiosum of some writers, and is best treated by snipping off its head, pressing out the contents of each sac, and applying a solution of potash one part, water two parts. When a great number of comedones, or black points, exist on the face or elsewhere, a steam-bath should be first taken, and subsequently the surface be smeared with the soap or washed with a solution of potash in glycerine. In this way the sebaceous matter is removed, and the skin may, by the after use of a wash of ether, alcohol and sulphur, be restored to its natural state.

Acne disseminata, which is an inflammation of the hair follicles, is generally caused by the formation of comedones, which, if not emptied, produce suppuration, and subsequent scars. The treatment must, therefore, be first directed to the removal of the comedones, which is best done by a wash of one part of potash to eight parts of water, or by use of the soap. Afterward, the sulphur lotion above mentioned may be used over night, and washed off the following morning with the potash solution. When the eruption is extensive, we rub in this soap, and leave it as a fomentation two or three days. When by this means the epidermis has been removed, the sulphur preparation should be applied.

Against *prurigo*, which is an incurable disease, returning always in the same individual, though often driven away by treatment, external applications are our only offensive weapons, and among these schmier-seife is perhaps the most reliable. It should be rubbed into the affected portions of the skin the first three days of the week twice daily, and be allowed to remain in contact, without washing away, the remaining four. This method, in connection on alternate weeks with daily morning dressings of cold water and cold baths, if continued for months will be found by far the most effectual in banishing this most distressing disorder.

In *psoriasis*, also, either this same mode of treatment is adopted by Hebra, or the use of the soap combined with applications of some form of tar, and with most excellent results. The internal administration of arsenic or cantharides he considers of questionable advantage.

It is in the treatment of *eczema*, however, in its varied forms, that the curative effect of applications of potash is most marked, and the mode of their employment is very simple. Of these, the following solutions are those generally used in the Vienna Klinik, which has done so much to simplify the treatment and classification of cutaneous diseases:

No. 1. Potassa pura one drachm, aqua Oi., as bath or fomentation.

No. 2. Potassa pura one drachm, aqua half-ounce, for circumscribed patches.

No. 3. Potassa pura two drachms, a caustic application.

In addition, potash in the form of schmier-seife and spiritus saponatus. Selection from these is made according to the extent and nature of the case. The two forms of eczema rubrum and eczema squamosom, into which the primary and acute stages generally run, are those which present themselves after the removal of the crusts, which is the first step in the treatment. This is easily affected by the application of warm oil and spiritus saponatus. We then for the first time can ascertain the condition of the skin, which is the seat of the disease. If the cutis is much thickened by exudation, as we find by lifting a fold, the severer remedies must be chosen. The excessive vascularity and enlargement of the capillaries, which cause the redness, heat, swelling and large effusion in eczema rubrum, must first be overcome by the constant application of cold water, either in form of fomentation or douche. Then solution No. 2 should be applied once or twice, by means of a hair pencil, or the soap be substituted thrice a day; using at the same time cold water to heal the excoriation they may perchance cause. Eczema on the face must often be treated by the caustic solution No. 3, quenching the subsequent reaction by cold water. Scars never follow its use. If the disease affect the whole surface of the limbs or body, it may be treated with saturating flannels with schmier-seife and applying them, covered with gutta percha cloth, to the patches. These should be removed twice daily for the first few days, after which they may be suffered to remain in contact for three or four days. This plan is to be continued till cure results, unless excoriations show themselves, in which case the cold water applications must be resumed. In the dry, scaly form, eczema squamosum, preparation's of tar are used with great benefit in most cases to hasten the desired end, and among these are the oleum cadini, or oil of cade, and the oleum fagi; which is the Russian tanning oil. These should be applied diluted with alcohol, and laid on very thin, for on the skin of some persons they may of themselves excite an eczema. Tar, when applied to the whole surface of the body, often causes strange symptoms, as vometing black matter, black urine and black diarrhœa; these secretions containing tar unchanged. Relapses may, it is true, follow this treatment, as they do any other, but it prevents the recurrence of the disease as effectually, and works more rapidly than all others. Chronic eczema of the scalp, for instance, which so often baffles the empirical attempts of a physician for months, may in this manner be cured in as many days, and this without the aid of internal medicine.

There is a saying common in Germany, that the schmier-seife is for the itch what the comb is for the louse; and all over its densely-populated soil, where the system of crowded barracks and wandering journeymen makes *scabies* as common among the lower classes as it once was here, this is the remedy used in its treatment, both in hospital and household. Upon its ready action are based the many quick cures, which boast to kill the disease in three or four hours. These methods, however, are not advisable, for often relapses follow, and eczema and excoriations are produced, which are far more difficult to heal than the original disease. The well-known plan adopted by Hebra is the following: The patient takes a warm bath, rubs thoroughly every part affected with a coarse flannel cloth saturated with schmier-seife, and, after washing off, smears the same parts with one of the following ointments or tinctures. This process is to be repeated every evening till itching ceases. Three baths are all that are generally allowed, else the skin becomes too much macerated and easily inflamed. Four days are usually sufficient to cure very bad cases even, and the circumscribed ravages of the animal may be stopped at once. The eczema, papules and pustules, which the parasites indirectly cause, are often not so easily dealt with, and require an after-treatment of their own. The following is the "Vienna Salve:" Sapo virid., axungia, each 3 parts; flor. sulph., pix liquida, each $1\frac{1}{2}$ parts; creta alb., 1 part. M. Hebra's own ointment is of similar composition:—Flowers of sulphur, oil of beech or of cade, each 6 ounces; schmier-seife, fat, each 16 ounces.—M. Chalk is added when necessary, to remove the epithelium more rapidly, as with soldiers or the great unwashed. In cases where fat cannot be used, he recommends the substitution of alcohol in the same amount. Either of these preparations may be used in connection with the soap, and the result of such treatment will be fully satisfactory to every one who may try it. The alkaline soap, when applied to burrow, produce at once an exudation into the same, which causes its immediate recognition. Its later effects are to dissolve the epithelium, and allow the sulphur to work directly upon the animals. The tar, or beech or juniper oils, are added to prevent the generation of excoriation or eczema by the excess of alkali and friction.

Whatever may be said about the ætiology of other cutaneous diseases in which vegetable parasites are detected, it is positively certain that pityriasis versicolor is caused by the fungus called microsporon furfur. This is no place to go into any description of the diseases, or to state how it differs from chloasmata, with which it is often confounded. No two diseases, however are more distinct. The intolerable itching which betrays the presence of the fungus, will cease at the death of the plant, which is easily caused in a short time by daily inunction with schmier-seife. Its effect upon the patches is wonderful.

It has been my object thus to show how valuable and general a remedy we have in this soap, and to endeavor to introduce it to the profession as an instrument both cheap and cleanly, and of sure promise certainly a long-looked-for desideratum in this class of diseases.

